# Supplementary material for: CD14 release induced by P2X7 receptor restricts inflammation and increases survival during sepsis
Source: eLife. 2020 Nov 2;9:e60849. doi: 10.7554/eLife.60849 (PMC7690950; doi:10.7554/eLife.60849)
Supplement: Supplementary file 1. — Table 2. Histopathology scoring (average of n = 3 animals/group). [file elife-60849-supp1.docx]

**Table1.** Demographics and clinical features of enrolled healthy volunteers and patients with intra-abdominal sepsis.

|  | **Non-septic** | | | **Septic patients** |
| --- | --- | --- | --- | --- |
| **N** | 10 | | | 9 |
| **Age**, mean (range) ± SD  *p* value *vs* septic group | 64.1 (61-66) ± 1.97  *p*> 0.05^ns^ | | | 67.77 (43-83) ± 10.04 |
| **Gender**, N (%)  Male  Female  *p* value *vs* septic group | 5 (50%)  5 (50%)  *p*> 0.05^ns^ | | | 4 (44.45%)  5 (55.55%) |
| **Associated pathologies,** N (%) | | | | |
| Diabetes mellitus Type II  Arterial hypertension  Brugada’s syndrome | 3 (30%)  2 (20%)  1 (10%) | |  | 3 (33.33%)  4 (44.44%)  0 |
| **Clinical data**  *(only for septic patients)* | | | | |
| **Initial septic focus,** N (%)  *Stomach*  *Colon*  *Sigmoid colon*  *Small intestine*  *Abdominal, unidentified* |  | | 1 (11.11%)  5 (55.55%)  1 (11.11%)  1 (11.11%)  1 (11.11%) | |
| **Isolated bacteria**, N (%)  *Escherichia coli*  *Streptococcus sp*  *Prevotella sp*  *Klebsiella sp*  *Proteus mirabilis*  *Candida albicans* |  | | 4 (44.44%)  4 (44.44%)  1 (11.11%)  2 (22.22%)  1 (11.11%)  2 (22.22%) | |
| **PCT**  mean (range) ng/ml ± SD |  | | 28.39 (1.08-100) ± 41 | |
| **CRP**  mean (range) mg/dl ± SD |  | | 37.62 (28.24-49.74) ± 7.17 | |
| **CREATININE**  mean (range) mg/dl ± SD |  | | 1.75 (0.73-3.25) ± 1.02 | |
| **ALBUMIN**  mean (range) g/dl ± SD |  | | 2.34 (1.8-2.9) ± 0.44 | |
| **UREA**  mean (range) mg/dl ± SD |  | | 92.78 (36-188) ± 55.66 | |
| **BILIRUBIN**  mean (range) mg/dl ± SD |  | | 1.58 (0.25-5.31) ± 1.74 | |
| **ASPARTATE TRANSAMINASE (AST)**  mean (range) U/L ± SD |  | | 123.8 (12-858) ± 276.5 | |
| **ALANINE TRANSAMINASE (ALT)**  mean (range) U/L ± SD |  | | 66.22 (10-443) ± 141.5 | |
| **ALKALINE PHOSPHATASE**  mean (range) U/L ± SD |  | | 83.11 (42-176) ± 42.05 | |
| **GAMMA GLUTAMYL TRANSFERASE**  mean (range) U/L ± SD |  | | 53.11 (8-189) ± 63.63 | |
| **SOFA**  mean (range) ± SD |  |  | 6.44 (3-11) ± 2.79 | |
| **APACHEII**  mean (range) ± SD |  |  | 17.33 (10-22) ± 4 | |
| **Mortality** N (%) |  |  | 1 (11.11%) | |

*ns*, no significant difference (*p*> 0.05); SD, standard deviation; Chi-square (𝜒^2^) test was used, except for age, where a one-way ANOVA test was used.

**Table 2**. Histopathology scoring (average of *n*= 3 animals/group).

|  | **Score** | | |
| --- | --- | --- | --- |
|  | **Liver*** | **Spleen**** | **Lung***** |
| C57 BL/6 sham | 2 | 0 | 0 |
| C57 BL/6 CLP 24 h | 5 | 8 | 6 |
| C57 BL/6 CLP 48 h | 4.7 | 8 | 7 |
| C57 BL/6 sham + A438079 | 2.7 | 0 | 0 |
| C57 BL/6 CLP 24 h + A438079 | 7.3 | 11 | 9 |
| C57 BL/6 CLP 48 h + A438079 | 7.3 | n/a | n/a |
| *P2rx7*^−/−^ sham | 3.7 | 0 | 0 |
| *P2rx7*^−/−^ CLP 24 h | 7.3 | 11 | 9 |
| *P2rx7*^−/−^ CLP 48 h | 8 | 11 | 9 |
| *P2rx7*^−/−^ CLP 24 h + rCD14 | 4.3 | 8 | 6 |

*Liver histological features: steatosis (0: none; 1: <20%; 3: >20%); ballooning (1: occasional; 4: marked); polymorphonuclear cell infiltration (1: none/mild; 3: severe); inflammation (0: none; 2: yes); fibrosis (0: none; 4: yes).

**Spleen histological features: unstructured spleen tissue (0: none; 3: yes); congestion of the white and red pulp (0: none; 3: yes); presence of apoptotic bodies (0: none; 5: yes).

***Lung histological features: alveolar congestion (0: none; 3: yes); alveolar thickening (0: none; 3: yes); polymorphonuclear cell infiltration (0: none; 3: yes); fibrosis (0: none; 5: yes).

n/a: not analysed**.**
