# Supplementary material for: CD14 release induced by P2X7 receptor restricts inflammation and increases survival during sepsis
Source: eLife. 2020 Nov 2;9:e60849. doi: 10.7554/eLife.60849 (PMC7690950; doi:10.7554/eLife.60849)
Supplement: Transparent reporting form [file elife-60849-transrepform.pdf]

eLIFE
1st Floor
24 Hills Road
Cambridge CB2 1JP, UK
P 01223 855340
W elifesciences.org
T @elife

eLife's transparent reporting form

We encourage authors to provide detailed information *within their submission* to facilitate the interpretation and replication of experiments.Authors can upload supporting documentation to indicate the use of appropriate reporting guidelines for health-related research (see EQUATOR Network), life science research (see the BioSharing Information Resource), or the ARRIVE guidelines for reporting work involving animal research.Where applicable, authors should refer to any relevant reporting standards documents in this form.

If you have any questions, please consult our Journal Policies and/or contact us: editorial@elifesciences.org.

Sample-size estimation

I. You should state whether an appropriate sample size was computed when the study was being designed
II. You should state the statistical method of sample size computation and any required assumptions
III. If no explicit power analysis was used, you should describe how you decided what sample (replicate) size (number) to use

Please outline where this information can be found within the submission (e.g., sections or figure legends), or explain why this information doesn't apply to your submission:

I. Referring to the sample size used in figures 1 and 2, we designed and performed the experiments based in our previous publications (de Torre-Minguela et al., 2016; Barberá-Cremades et al., 2016). This information can be found in figure legends, where each dot represents the result obtained by the use of a single bone marrow-derived macrophage culture obtained from one single mouse. Concerning figures 3, 4, 5 and 6, the animals/human patients sample size was determined based in scientific literature (Csóka et al., 2015; Santana et al., 2015; Martínez-García et al., 2019). This information can be found in figure legends, where each dot represents one independent mouse/human patient. The sample size was determined taking into account that we want to obtain a statistical power of 95% and a significant difference between the results of 0.05% using a restrictive nonparametric test.
II. For the statistical analysis of our results we have used the nonparametric Mann-Whitney *U* test assuming that our work is done in independent sample groups, we have independence of observations and our samples are not normally distributed. Specifically, for figure 6 a-b we have used the nonparametric Log-rank (Mantel-Cox) test to compare survival distributions of two samples. This information can be found in the figure legends, as well as in the Material & Methods section.
III. No explicit power analysis was used. For figures 1 and 2, as the *in vitro* approach was exploratory at the beginning and the final results were unknown, we determined our sample size with n>3 initial replicates, when a significant difference was found, then we used different experimental approaches to investigate the mechanisms that in some cases increases the n number of basic control experiments. For figures 3, 4, 5 and 6 we based our *in vivo* sample size accounting for the 3Rs principles: replacement, reduction and refinement and previous publications, as stated above. The sample size can be found in figure legends.

Replicates

I. You should report how often each experiment was performed
II. You should include a definition of biological versus technical replication

III. The data obtained should be provided and sufficient information should be provided to indicate the number of independent biological and/or technical replicates
IV. If you encountered any outliers, you should describe how these were handled
V. Criteria for exclusion/inclusion of data should be clearly stated
VI. High-throughput sequence data should be uploaded before submission, with a private link for reviewers provided (these are available from both GEO and ArrayExpress)

Please outline where this information can be found within the submission (e.g., sections or figure legends), or explain why this information doesn't apply to your submission:

I. The experiments were performed in sets of animals that always included all different groups analyzed (different genetic background, controls, sham, CLP, treated, non-treated, etc.). The experiments were repeated to minimize variability and gain statistical power respecting the 3Rs principles.
II. A biological replicate accounts for the results obtained from one independent single mouse generating only one data set (including the bone-marrow derived macrophages referred in figures 1 and 2) or individual human patient sample. A technical replicate accounts for the results obtained using a single mouse or human patient sample with the same treatment and generating two or more data sets.
III. In this paper, we have exclusively used biological replicates. The number can be found in figure legends.
IV. Once we had all data and before performing the statistical analysis, to identify possible outliers we used the ROUT method (robust regression followed by outlier identification) with Prism (GraphPad). Q value was set to 1% in order to control the False Discovery Rate, meaning that less than 1% of the "statistically significant" findings will be false positives.
V. See Table 1 below.
VI. Do not apply.

Table 1			
**Figure**	**Explanation**	**Inclusion criteria**	**Exclusion criteria**
1 and 2	*In vitro* experiments from bone marrow-derived macrophages.	-	**Extracellular LDH value as measurement of cell viability**. When negative or positive controls wells presented a high cell death.
3*, 4, 5 and 6	*In vivo* experiments involving cecal ligation and puncture (CLP)-procedure.<br><br>* 3a. *In vitro* experiments with human samples.	The animals underwent successful CLP surgery as stated at Rittirsch et al., 2009.<br><br>* **Septic patients**. Intra-abdominal sepsis with multiple organ dysfunctions (specific inclusion criteria available in material & methods).	**Weight.** If an animal lost more than a 20% of its body weight. **Prematurely dead**. **Animal welfare.** If an animal did not accomplish the welfare criteria, was euthanized. **Outlier identification** using the ROUT method.<br><br>* **Healthy volunteers** with associated-pathologies.

Statistical reporting

- Statistical analysis methods should be described and justified
- Raw data should be presented in figures whenever informative to do so (typically when N per group is less than 10)
- For each experiment, you should identify the statistical tests used, exact values of N, definitions of center, methods of multiple test correction, and dispersion and precision measures (e.g., mean, median, SD, SEM, confidence intervals; and, for the major substantive results, a measure of effect size (e.g., Pearson's r, Cohen's d)
- Report exact p-values wherever possible alongside the summary statistics and 95% confidence intervals. These should be reported for all key questions and not only when the p-value is less than 0.05.

Please outline where this information can be found within the submission (e.g., sections or figure legends), or explain why this information doesn't apply to your submission:

All the information accounting for statistical reporting is stated in material and method section, as well as in figure legends.
All the graphs represent raw data (each dot represents the value of an individual animal or experiment) and p-value is represented in all matter questions.

(For large datasets, or papers with a very large number of statistical tests, you may upload a single table file with tests, Ns, etc., with reference to sections in the manuscript.)

Group allocation

- Indicate how samples were allocated into experimental groups (in the case of clinical studies, please specify allocation to treatment method); if randomization was used, please also state if restricted randomization was applied
- Indicate if masking was used during group allocation, data collection and/or data analysis

Please outline where this information can be found within the submission (e.g., sections or figure legends), or explain why this information doesn't apply to your submission:

Experimental group allocation does not apply to our submission, as all the experimental groups are from homogenous cell cultures wells plated form the same cell differentiation plates on the same day, or from matched age/sex animals. Therefore, the initial application of the different treatments are not conditioned to group allocation.

Additional data files ("source data")

- We encourage you to upload relevant additional data files, such as numerical data that are represented as a graph in a figure, or as a summary table
- Where provided, these should be in the most useful format, and they can be uploaded as "Source data" files linked to a main figure or table
- Include model definition files including the full list of parameters used
- Include code used for data analysis (e.g., R, MatLab)
- Avoid stating that data files are "available upon request"

Please indicate the figures or tables for which source data files have been provided:

eLife Sciences Publications, Ltd is a limited liability non-profit non-stock corporation incorporated in the State of Delaware, USA, with company number 5030732, and is registered in the UK with company number FC030576 and branch number BR015634 at the address 1st Floor, 24 Hills Road, Cambridge CB2 1JP | August 2014

eLIFE
1st Floor
24 Hills Road
Cambridge CB2 1JP, UK
P 01223 855340
W elifesciences.org
T @elife

All the graphs presented in the figures include raw data (each dot represents the value of an individual animal or experiment). We also supply source data for main figures.

eLife Sciences Publications, Ltd is a limited liability non-profit non-stock corporation incorporated in the State of Delaware, USA, with company number 5030732, and is registered in the UK with company number FC030576 and branch number BR015634 at the address 1st Floor, 24 Hills Road, Cambridge CB2 1JP | August 2014
